# Tackling structural stigma: a systems perspective

**DOI:** 10.1002/jia2.25924

**Published:** 2022-07-12

**Authors:** Graham Brown, Daniel Reeders, Aaron Cogle, Brent Allan, Chris Howard, John Rule, Susan Chong, Deborah Gleeson

**Affiliations:** ^1^ Centre for Social Impact University of New South Wales Sydney New South Wales Australia; ^2^ Australian Research Centre in Sex, Health and Society La Trobe University Melbourne Victoria Australia; ^3^ Collaboration for Evidence, Research and Impact in Public Health Curtin University Perth Western Australia Australia; ^4^ School of Regulation and Global Governance Australian National University Canberra Australian Capital Territory Australia; ^5^ National Association of People Living with HIV Australia Sydney New South Wales Australia; ^6^ Qthink Consulting Melbourne Victoria Australia; ^7^ Formerly of Living Positive Victoria Melbourne Victoria Australia; ^8^ Queensland Positive People Brisbane Queensland Australia; ^9^ School of Population Health University of New South Wales Sydney New South Wales Australia; ^10^ School of Psychology and Public Health La Trobe University Melbourne Victoria Australia

**Keywords:** community, peer, PLHIV, stigma, structural, systems

## Abstract

**Introduction:**

Structural stigma in the global HIV response is a “moving target” that constantly evolves as the epidemic changes. Tackling structural stigma requires an understanding of the drivers and facilitators of stigma in complex community, policy and health systems. In this paper, we present findings from a study adopting a systems perspective to understand how to tackle structural stigma via the Meaningful Involvement of People with HIV/AIDS (MIPA), while highlighting the challenges in demonstrating peer leadership from people living with HIV (PLHIV).

**Methods:**

Through a long‐term ongoing community‐research collaboration (2015–2023), the study applied systems thinking methods to draw together the insights of over 90 peer staff from 10 Australian community and peer organizations. We used hypothetical narratives, affinity methods and causal loop diagrams to co‐create system maps that visualize the factors that influence the extent to which peer leadership is expected, respected, sought‐out and funded in the Australian context. We then developed draft indicators of what we should see happening when PLHIV peer leadership and MIPA is enabled to challenge structural stigma.

**Results:**

Participants in the collaboration identified the interactions at a system level, which can enable or constrain the quality and influence of PLHIV peer leadership. Participants identified that effective peer leadership is itself affected by structural stigma, and peer leaders and the programmes that support and enable peer leadership must navigate a complex network of causal pathways and strategic pitfalls. Participants identified that indicators for effective PLHIV peer leadership in terms of engagement, alignment, adaptation and influence also required indicators for policy and service organizations to recognize their own system role to value and enable PLHIV peer leadership. Failing to strengthen and incorporate PLHIV leadership within broader systems of policy making and health service provision was identified as an example of structural stigma.

**Conclusions:**

Incorporating PLHIV leadership creates a virtuous cycle, because, as PLHIV voices are heard and trusted, the case for their inclusion only gets stronger. This paper argues that a systems perspective can help to guide the most productive leverage points for intervention to tackle structural stigma and promote effective PLHIV leadership.

## 1 INTRODUCTION

Despite advancements in HIV care and prevention, HIV stigma and discrimination continue to undermine quality of life for people living with HIV (PLHIV) [1]. In order for multi‐level interventions to meaningfully reduce stigma [2, 3], we need to understand stigma at a system level. This was reinforced in a recent consensus statement calling for “health systems and the people who work within them [to] recognize and work to eliminate the multiple forms of structural discrimination that undermine the health of PLHIV” [4].

The Greater and Meaningful Involvement of the People with HIV/AIDS (GIPA/MIPA) [5, 6] has long been recognized as central to an effective response to HIV [7, 8], including in strategies and policies to tackle systemic HIV stigma [9, 10]. GIPA/MIPA is often applied through the involvement of networks of PLHIV, community‐based organizations, key population networks and civil society [8]. In turn, opportunities for peer leadership are often enabled by mobilizing and strengthening communities most affected by HIV. This includes peer‐ and community‐led (PCL) responses by gay and bisexual men, people who use drugs, sex workers and PLHIV, navigating highly stigmatized and political contexts around sex, sexuality and drug use. These peer‐led responses operate through organizations established and governed by their communities. PLHIV have played a critical role in PCL programmes, including peer support, health promotion, community mobilization, leadership and policy advocacy [11, 12]. Strengthening community systems and PLHIV peer leadership is increasingly recognized in policy and strategy documents as critical to impactful responses to HIV stigma [8, 13, 14, 15, 16].

This paper presents findings from the W3 (Understanding What Works and Why in Peer Based Programmes in HIV and Hepatitis C) project, in which we used complex systems theory and methods to investigate the factors that influence the extent to which peer leadership is expected, respected, sought‐out and funded in Australia.

The aim of this paper is to better understand how to tackle structural stigma via the Meaningful Involvement of People with HIV/AIDS (MIPA), while highlighting the challenges that must be navigated to demonstrate effective peer leadership in the process.

The W3 project is an ongoing long‐term collaboration (stage 1: July 2015–June 2017; stage 2: July 2017–December 2019; and stage 3: January 2020–June 2023). The project sought to deepen understanding of the socio‐ecological system(s) in which PCL programmes operate in Australia. This report discusses the first two stages of this project, while the final stage is currently underway.

## 2 METHODS

The project uses a qualitative research method adapted from systems thinking [17]. The method and its adaptation are described in Reeders and Brown [18]. Systems thinking views the world as composed of dynamic, interactive networks and systems. It aims to recognize patterns in their overall function, rather than attributing causal effects to their individual components. In this research, we are identifying patterns of interaction that recur often enough, and exert enough influence, to make them worth mapping.

The W3 Project responded to the need voiced by Australian PCL organizations to improve scholarly and policy‐maker understandings of what works and why in peer‐based HIV and hepatitis C prevention and health promotion. In response to recommendations from a subsequent scoping study, conducted in consultation with the community sector [19], a collaboration was formed to improve the evidence base regarding the role that PCL programmes play in the overall HIV and hepatitis C prevention system.

The W3 collaboration (Table 1) formalized existing relationships among HIV and hepatitis C community organizations in Australia. All relevant community organizations were invited to participate at each stage of the study and took part according to their capacity to commit staff time. While organizations self‐selected to be part of the project, the study includes organizations of diverse size, location and jurisdictional scope (state/territory and national).

**Table 1:** The W3 collaboration

W3 Project: Understanding what works and why in peer‐based and peer‐led programmes in HIV and hepatitis C
Australian Federation of AIDS Organisations (the national body for the community‐based response to HIV, whose members include peer‐ and community‐led organizations)
Australian Injecting and Illicit Drug Users League (the national body for peer‐based drug user organizations)
Harm Reduction Victoria (peer‐based drug user organization)
Living Positive Victoria (PLHIV peer‐based organization)
National Association of People Living with HIV/AIDS (national peer PLHIV organization)
Positive Life New South Wales (PLHIV peer‐based organization)
Queensland Positive People (PLHIV peer‐based organization)
NSW Users and AIDS Association (peer‐based drug user organization)
Scarlet Alliance – Australian Sex Workers Association (national peer‐based sex worker organization)
Victorian AIDS Council (community‐ and peer‐based organization with services for and by gay and bisexual men and PLHIV)
Western Australian Substance Users Association (peer‐based drug user organization)
In Australia, “community‐based” and “peer‐based” are the dominant organizational descriptors. These organizations were established by the communities most affected by HIV from the mid‐1980s, and their governance is based within their communities. For a summary of the history of the community response in Australia, see [20]

Abbreviations: NSW, New South Wales; PLHIV, people living with HIV.

Peer leaders who volunteered to participate from the participating organizations (Table 1) were members of communities of PLHIV, gay and bisexual men, people who use drugs and sex workers. Most peer leaders had multiple intersections across these and other communities impacted by HIV. Most were paid peer staff who had worked in peer organizations for over 3 years, some over 10 years, in a variety of service delivery, outreach and advocacy roles. Organizations in the W3 collaboration and individual participants from each organization provided signed informed consent to participate in workshops at each stage.

Consistent with the system mapping methodology [17, 18] as a form of research co‐production, the project followed a dynamic and iterative process in constant conversation between peer practitioners and the research team to develop “system maps.” The system maps and their accompanying text descriptions are the qualitative data analysed in this paper.

The identity of the organizations was not confidential (Table 1). At the request of the peer organizations, workshops in stage 1 and stage 2 were not recorded and transcribed to ensure confidentiality of the participating individuals’ views and experiences, and confidentiality of the discussion of the detailed specific examples of how organizations navigated stigmatized, criminalized and political environments. The project was provided ethics approval by the La Trobe University Human Research Ethics Committee (Approval No: FHEC14/155).

### 2.1 Stage 1—Systems mapping

Stage 1 (July 2015–June 2017) involved three case studies at different levels of the “prevention system,” including a needle and syringe exchange programme working in frontline service delivery; a social network‐based health promotion initiative targeting sexually adventurous men; and an HIV‐positive peer advocacy strengthening initiative. The three case studies were chosen through consultation within W3 collaboration to ensure a diversity of peer contexts and locations within the relevant HIV and hepatitis C prevention systems, as well as geographic diversity in Australia.

We conducted 18 workshops, ranging from 1 to 2 days each, with 10 PCL organizations across Australia (Table 1). Each workshop featured between 1 and 4 organizations. We drew on the experience and perspectives of more than 90 peer practitioners working in outreach, community development, workshop facilitation, policy reform and leadership, management and governance. All 10 partner organizations involved PLHIV within peer‐led programmes. However, four organizations were specifically PLHIV peer‐led organizations with strong PLHIV leadership roles. These included three state‐based organizations and one national peak organization, which represents state‐based organizations in national advocacy.

Workshops used hypothetical narratives, affinity methods and digital drawing tools to develop causal loop diagrams, which visualize the feedback loops that emerge between variables and processes in an ongoing system [17]. This method helped us to identify and understand the complex relationships between all the moving parts of a community and policy system, drawing on complexity science to conceptualize how interactions among actors can generate emergent structures (such as networks, cultures and communities) and effects (including overall prevention efficacy) [21, 22, 23].

To validate the maps, the workshops explored system dynamics by participants selecting an issue from their practice and a starting place on the map, and then following the pathways laid out by arrows and items, identifying and discussing the implications for that issue. The workshops tested hypotheticals, asking “if this element suddenly stopped working, what might happen elsewhere in the system?” Participants drew on their peer work experience and the maps were refined where required to reflect the participant's experience [18]. These implications identified through the system mapping formed part of the system descriptions.

**Figure 1:**
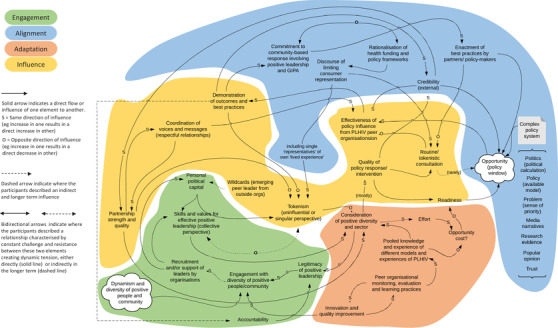
PLHIV peer leadership system map. Abbreviation: GIPA, Greater Involvement of People Living with HIV/AIDS.

**Figure 2:**
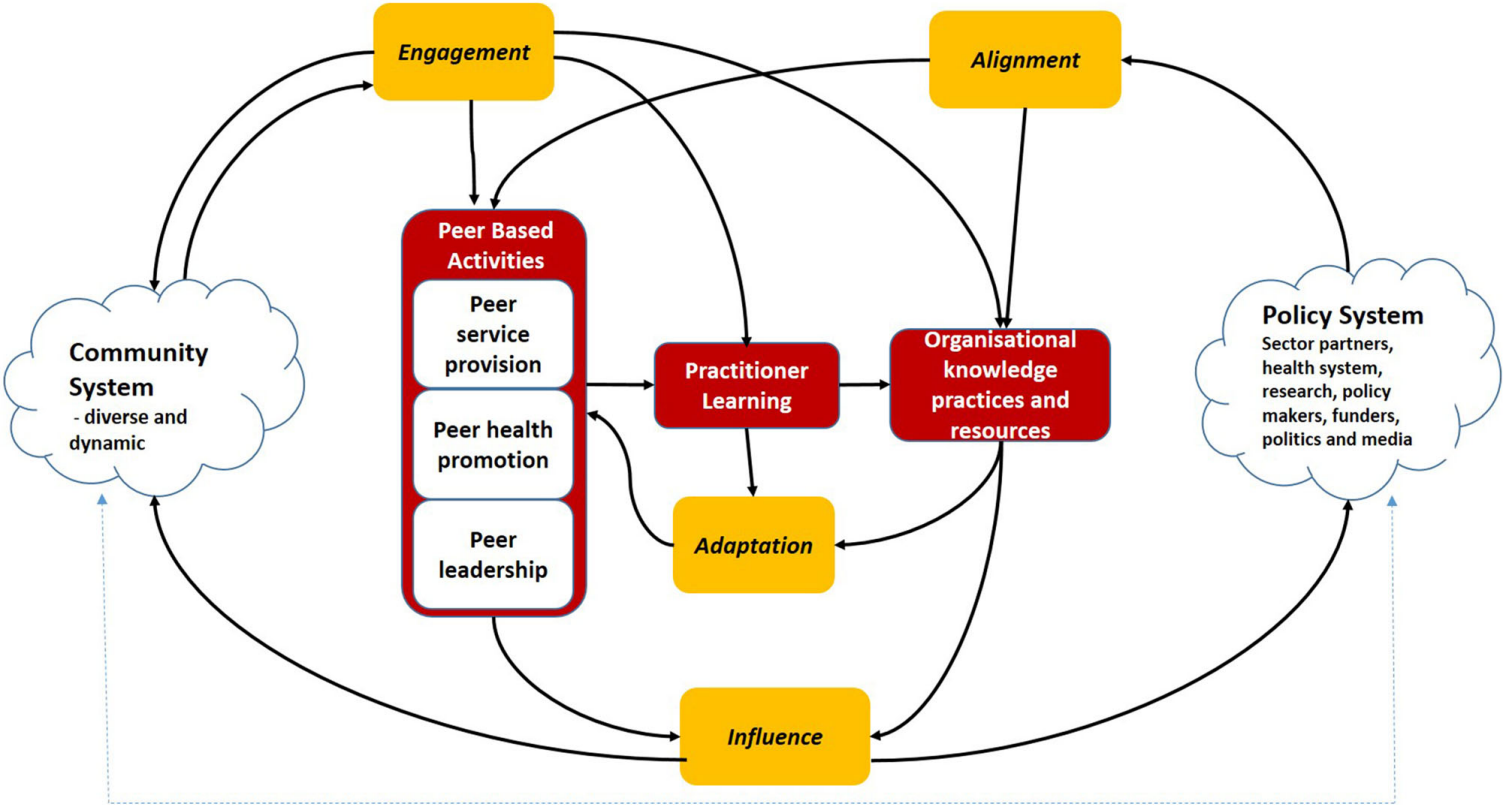
W3 framework.

Drawing on the realist evaluation work of Pawson and Tilley [24], we analysed the full set of complex system maps (for an example, see Figure 1) to identify the key underlying functions that occurred across all the system maps and which enable peer‐led responses to be effective and sustainable in continually changing community and policy environments (W3 framework, Figure 2). The methodology of stage 1 has been described in detail elsewhere [18, 25, 26].

**Table:** 

Element	Definition
Community system	The community system includes the networks and cultures the programme engages with, and the processes of interaction and change that are taking place within them.
Policy system	The policy system includes sector partners and stakeholders, funders, policy‐makers, health system, surveillance and research, politicians, news media, and other organisations which interact with the peer programme and its communities.
Engagement, Alignment, Adaptation and Influence	Functions that are required within the system for peer‐led programmes to be effective and sustainable in a constantly changing environment.
Peer based activities	Different kinds of peer based approaches that depend on practitioners having and using peer skill‐the ability to combine personal experience and real‐time collective understanding to work effectively within a diverse community.
Practitioner learning	Staff and volunteers in peer based programmes pick up insights from clients and their own networks, and in their practice over time they develop, test and refine mental models of their environment.
Organisational knowledge practices	Organisational management values and learns from the analysis of insights from peer practitioners, supporting the adaptation process and sharing with stakeholders in the community and policy system.
Arrows	Flows of knowledge or causal influence that constitute the programme as a system.

### 2.2 Stage 2—Development of monitoring, evaluation and learning framework (July 2017–December 2019)

In stage 2 (July 2017–December 2019), the project conducted further workshops with W3 collaboration members (more than 35 participants), drawing on the W3 framework to develop tailored indicators for the role, quality and influence of, among other things, peer leadership in their local policy‐making and health service provision system [27, 28]. These indicators were then trialled and refined in practice within two peer organizations (one PLHIV peer organization and one people who use drugs peer organization). The approach and outcomes of this stage have been described elsewhere [29, 30, 31, 32]. Further details are available from www.w3project.org.au.

## 3 RESULTS

Participants in all the system workshops identified that peer‐led responses operate within and mediate between complex and constantly changing community and policy system elements. In this paper, we focus on the results of the PLHIV peer leadership case study, which most clearly illustrates the factors that can either diminish or reinforce the valuation of peer leadership in policy‐making and service provision. This section focuses on the feedback loops that relate most directly to structural HIV stigma, and identifies their long‐term implications based on workshop discussions held during the co‐production process.

### 3.1 Orientation to the PLHIV peer leadership map

The PLHIV peer leadership system map in Figure 1 illustrates the relationships and processes that constitute the Australian HIV policy‐making and health service provision system from the perspective of a national policy advocacy initiative known as “Poz Action.” Participants described how a range of frontline programmes, including peer education, support, outreach, mobilization and advocacy, are interconnected and nested within this broader system. The map developed by the participants visualizes a matured, organized and community‐based response to HIV that navigates many pitfalls that can lead to undervaluing peer leadership.

In order to simplify and operationalize the three complex system maps, we developed the W3 framework (Figure 2) as a mid‐level theory that presents PCL programmes as *contextual interventions* that operate within and between two interrelated and continually changing complex systems: affected communities (left cloud) and policy system (right cloud). The framework highlights four key functions—engagement, alignment, adaptation and influence—that must be happening for PCL programmes to maintain relevance and influence as these systems change and evolve around them. These four key functions have been used to shade relevant areas of the PLHIV leadership map in Figure 1:

- engagement (green) concerns the embedding of the peer‐led programme within affected communities;
- alignment (blue) reflects its relationship with the HIV sector and its policy context;
- adaptation (orange) refers to processes for adapting programmes according to what they learn from engagement and alignment;
- influence (yellow) reflects the outcomes of programme activities in the community and policy systems.

In what follows, we use these functions to describe selected system dynamics identified by participants through the workshops—key feedback loops and longer causal pathways—and the identified implications for the valuation of peer leadership in the policy system. In the discussion below, italicized text refers to textual items on the system map (Figure 1).

### 3.2 Function 1: Engagement within the PLHIV community system

Systems thinking reminds us that not only is the environment constantly changing, but changes are often emergent and, therefore, hard‐to‐predict. Workshop participants identified that PLHIV peer organizations are uniquely positioned to notice cues and patterns in their community and, if they capture and use this knowledge effectively, they can demonstrate peer leadership in policy systems that may struggle to accurately predict the impact of policies on PLHIV. Conversely, failing to use this knowledge can lead to de‐valuation of peer leadership.

Participants described that effective PLHIV peer leaders draw on their own experiences and social networks. But they also utilize insights from PLHIV peer programmes that engage with a broader range of PLHIV identities, needs and experiences. Our participants reported that this enables peer leaders to develop and constantly refine a *collective perspective* on the issues affecting different people and groups in the PLHIV community. This collective perspective distinguished charismatic individual advocacy from effective peer leadership in policy reform spaces.

Secondly, high‐quality engagement with the diversity of the PLHIV community meant having a mix of formal and informal processes to *recruit and/or support PLHIV peer leaders*. Formal process may include taking part in reference groups or policy committees, or being elected to organizational board or sub‐committees. This formal process supports the PLHIV leader to develop *personal political capital* as well as *skill and values for effective positive (PLHIV) leadership*. Informal processes were equally important and included identifying latent potential for leadership among clients and contacts, and then mentoring them or connecting them with opportunities for advocacy. This may be through identifying people with experience of an emerging issue, or who bring a new or underrepresented perspective, or who have charisma or *personal political capital* capable of capturing the attention of community or policy systems.

Effective PLHIV leaders were contrasted by our participants with “*wildcards*” who have significant *personal political capital* based on a strong narrative and personal charisma, but whose advocacy is not supported by a collective perspective and insight into the broader PLHIV community. If PLHIV leadership was characterized with this type of advocacy, participants noted the risk of tokenistic inclusion leading to reduced trust in PLHIV voices in the future. Particular kinds of peer leadership can either challenge or reinforce ongoing structural stigma in policy‐making networks and systems.

### 3.3 Function 2: Alignment between health system and peer‐led responses

The alignment function allows peer‐led responses to pick up insights into changes in the policy system (such as health services, legal and regulatory, epidemiology and social research), as well as gauge their own influence within that system. However, as illustrated in the alignment domain in Figure 1, participants identified that the *rationalization of health funding and policy frameworks* can negatively influence a health system's *commitment to community‐based response involving PLHIV leadership*. This tension can promote tokenistic inclusion of PLHIV, decreasing the opportunity, credibility and capacity for PLHIV peer organizations and leadership to influence policy at a time when the system most needs their community insights and engagement. This also affects the capacity for the peer leadership to gain early insights into emerging health system changes and to adapt programmes in response. When peer leadership is excluded from policy networks, the quality of alignment suffers, making it harder to engage in the kind of policy advocacy that demonstrates the value of PLHIV peer leadership—thus reinforcing structural stigma.

### 3.4 Function 3: Adaptation to changing community, policy and health system contexts

As illustrated in the W3 framework (Figure 2), to drive adaptation in response to the changes occurring within communities, health systems and policy, participants identified that PLHIV peer leaders needed to draw insights from both their communities (engagement) and from their policy environment (alignment). These insights must be used to guide adaptation across the range of programme activities, including peer service delivery, health promotion and peer leadership.

Participants also identified that for insights to be translated into adaptations, the PCL programmes and their partners in the HIV response need to understand these insights and support continuous and rapid adaptation. This included ensuring the formal and informal leadership opportunities for PLHIV responded to the emerging barriers for PLHIV to participate, whether due to stigma, perceived lack of professional experience or minority status.

For example, as illustrated in the adaptation domain in Figure 1, interactions between innovation and quality improvement; organizational monitoring, evaluation and learning practices; and pooled knowledge and experience of different models and experiences of PLHIV were identified by participants to all contribute to the consideration of positive [PLHIV] diversity and sector perspectives in policy‐making forums and spaces. These factors all affect the influence domain as well, represented on the map by the quality of the policy response/intervention as well as the peer organizations’ readiness to respond to shifting opportunities for policy reform. This demonstrates how quality adaptation is essential for consistently producing policy interventions that demonstrate effective peer leadership and challenge structural stigma.

### 3.5 Function 4: Influence on the health and policy system

Insights from PLHIV peer‐led responses can be the broader HIV sector's only source of close‐to‐real‐time knowledge about emerging issues and unintended consequences for PLHIV in rapidly changing and hard‐to‐predict socio‐ecological environments. Examples included the emergence of stigma towards PLHIV unable to reach undetectable viral load, the emergence of “PrEP 4 PrEP” sexual sorting based on use of pre‐exposure prophylaxis and how changing migration conditions impact upon willingness to access HIV care. Given this unique perspective, findings from formal and informal *community monitoring* [33] were identified as an influential strategic asset for the broader HIV response. This is a key pathway for demonstrating the value and effectiveness of peer leadership.

A second key pathway was identified as requiring careful navigation. Consistent with Kingdon [34], participants described that policy reform requires playing the “long game.” This poses *credibility* of peer leadership as an ongoing concern. PLHIV leadership must be seen and endorsed as credible, not just within policy‐making spaces, but also within the HIV sector and associated policy and health networks, in order to influence policy‐making. Participants describe that often, policy and health system influence is *leveraged* by involving other policy actors, who can advocate for and amplify positions carefully developed through peer leadership. Similarly, demonstrating leadership in whole‐of‐sector policy responses improves alignment—the ability of peer leadership to tread the shifting sands of policy reform. This requires more than consultation: it depends on relationships and trust and a sectoral culture that values HIV‐positive peer leadership in policy‐making.

Experienced PLHIV leaders in our study reported their influence on different issues was not independent or “once‐off,” but rather depended on having ongoing and demonstrable engagement within the PLHIV community, as well as a track record of high quality, timely and relevant previous contributions to policy‐making or health service reform. Participants identified that accountability, credibility and institutionalized stigma were constantly negotiated within both community and policy systems. As illustrated in Figure 1, this analysis suggested that the *quality of policy responses* and *effectiveness of policy influence* are only indirectly related. Policy influence is moderated by credibility and stigma, commitment to a community response, trust and past performance. Thus, the system map illustrates valuation as a key leverage point [35] for improving GIPA/MIPA and challenging the structural stigma that persists when PLHIV are excluded from policy‐making or health system reform spaces.

Our participants identified a second crucial dynamic in the prevalence of *tokenistic inclusion*. Multiple causal loops in Figure 1 feed into and out of tokenism (centre of diagram), reflecting its central role in a system where PLHIV inclusion is mandatory but listening to PLHIV voices remains optional. Participants identified the quality and impact of PLHIV peer leadership's influence in the policy and health system was mediated by the tension between a sectoral *commitment to a community‐based response and GIPA* and the health system‐level pressures (such as *rationalization of health funding and policy* and *discourse of consumer representation*).

The latter can shift the system towards tokenism, the inclusion of PLHIV in policy processes without meaningful influence on policy outcomes. In this context, participants described that the appearance of PLHIV “involvement” is what matters, and a policy or health organization may not inquire too closely into whether the position being advanced is based on *peer skill, collective perspective* and the *consideration of positive [PLHIV] diversity and sector perspectives*. Structural stigma thrives when the messy reality of the PLHIV lived experience can be treated as unwelcome complications rather than essential considerations. Tokenistic inclusion can lead to relying on individual perspectives at the cost of collective perspectives or balancing community and sector interests. This impacts on the quality of advice and also the credibility of PLHIV participation. In Figure 1, we have used bidirectional arrows to illustrate these relationships described by participants as characterized by constant challenge and resistance between elements, creating a dynamic tension.

### 3.6 Indicators of effective PLHIV peer leadership

In stage 2, we drew on the PLHIV peer leadership system map (Figure 1), the W3 framework (Figure 2) and the collaborative pilot work, to identify indicators for effective engagement, alignment, adaptation and influence. Table 2 presents the indicators for the quality and impact of PLHIV peer leadership in ways that can challenge structural stigma.

**Table 2:** Examples of W3 framework indicators for meaningful involvement of people living with HIV

	Quality actions/process indicators	Indicators of impact towards meaningful involvement
Engagement indicators	Diverse PLHIV peer leaders are regularly identified, recruited and supported from across peer programmes PLHIV leaders demonstrate the use of personal experience, cultural knowledge and evidence informed insights to communicate and work effectively with community (i.e. peer skill) Structures, processes and opportunities are in place to support peer workers to learn from each other's insights and maintain a current overall understanding of their diverse communities	PLHIV recognize the peer organization as an important part of, participant in and resource to their community Increasing willingness of PLHIV community to engage in sector consultation and leadership opportunities
Alignment indicators	The peer organization actively seeks out opportunities for policy contributions and advocates for creating safer and effective ways for community members to participate in the health and policy sector's response PLHIV peer leaders communicate with policy and sector partners to improve each other's understanding of responses to emerging issues	The PLHIV peer organization is informed about changes within the health system and policy environment and invited to assess how they might affect its communities and/or its work Key players from the broader health sector and policy environment recognize the peer organization as credible, trustworthy and an essential partner in the overall public health response Policy and sector allies publicly demonstrate they value the advice from PLHIV peer leadership and their commitment to a community‐based response
Adaptation indicators	The peer organization's practices are guided by peer knowledge and insights The peer organization draws on engagement with PLHIV, evaluation of peer programmes and partnerships with the sector to develop evidence‐based responses	Peer leaders demonstrate the ability to apply a peer lens to update their collective perspective of the community and policy systems and pre‐empt the implications of changes in the system The peer organization draws on community and sector insights to improve policy advice
Influence indicators	Policy and health services Peer leadership is enabled to draw on strength of engagement, alignment and peer skill to respond to opportunities for policy participation and influence Peer leadership is enabled to be responsive to opportunities for policy participation and provide policy advice when needed The peer organization maintains control over the use and interpretation of the information they share with external stakeholders	Policy and health services Policy makers and health services seek out the advice of PLHIV peer leaders based on quality of past advice The policy and health system demonstrates that it values the peer approach and has trust in the quality of the insights it generates The peer organization can demonstrate buy‐in from stakeholders to advance community needs and enhance the HIV continuum of care
	Community The organization supports peer leaders to build their confidence, skill and experience in community and personal advocacy Expanding community influence is reflected in new and diverse PLHIV engaging in peer leadership opportunities	Community Coordinated peer leadership results in a strong collective community voice that contributes to policy recognition of diverse needs and experiences within the community PLHIV community looks towards PLHIV peer leadership to provide insights based in the reality of their shared lives

## 4 DISCUSSION

Findings from the W3 project show the pathways—and pitfalls—that must be navigated in order to demonstrate and promote effective PLHIV leadership, and to challenge the structural stigma that devalues peer voices and perspectives in the Australian HIV prevention and health promotion system. In particular, it highlights the complex interplay of factors that can either diminish or reinforce the positive valuation of peer leadership in policy‐making and health service provision. It demonstrates that PLHIV inclusion involves a network of actors, relationships and practices, not simply placing a person living with HIV on a committee.

Across all three Australian case studies in stage 1, the study mapped a process by which insights from the daily realities of sex and drug use are captured through peer service provision, shared within peer organizations and re‐packaged with relevant insights from research or policy other inputs into formats that were able to be understood and recognized in policy forums and research. This process has been described as “translation” [36]. This can contribute to more effective and responsive policy‐making, in part because this process offers close to real‐time knowledge of rapidly changing situations. However, it depends on valuing the HIV‐positive voice in policy.

Structural stigma can occur when peer insights and the positive voice are not valued. Sub‐optimal policy, made when peer leadership is not respected, has wide‐ranging effects as it is put into practice across the system. But secondly, stigma directly affects the structure of that system itself, as it marginalizes or excludes particular actors from exerting influence. Recognizing these two senses in which stigma is structural helps identify possible solutions. It is not enough simply to include peer voices in decision making. Organizations that facilitate the inclusion of peer voices also need to ensure their involvement demonstrates visible peer leadership—and the benefits in terms of quality policy‐making.

The systems perspective provides an insight into the system impact of a model of peer leadership that resists the domination of a single, convenient or tokenistic narrative, but that values and incorporates the diverse and evolving experience of PLHIV. It highlights that both policy‐making and health service provision consist of systems—and so systems perspectives and methods are essential for understanding how effective peer leadership can be supported by and exert influence within them.

For example, interventions to improve PLHIV engagement across the HIV continuum of care increasingly recruit PLHIV as peer navigators [37, 38, 39]. Peer navigators use their lived experience to help other PLHIV navigate complex systems of care and support provision, and thus build up knowledge of those systems’ workings and shortcomings. As health systems endeavour to reduce stigma and enhance the continuum of care, these peer navigators gain unique insights into the experiences of their diverse peers, building a collective understanding of the effectiveness of the changes in the health service system as they occur. For this knowledge to be shared and influential in health system reform, these insights and navigators’ potential for policy leadership must be valued and resourced within the health and policy system.

The indicators in Table 2 describe what we should be seeing happening with effective engagement, alignment, adaptation and influence. They provide a starting point for understanding how GIPA/MIPA can reduce stigma and enhance the HIV continuum of care. The W3 framework and indicators help guide answers to the question: how do we *know* if we are demonstrating effective PLHIV leadership in ways that challenge structural stigma?

The findings illustrate that as PLHIV peer programmes and peer leadership, we should be capturing insights from engagement and alignment; we should be able to point to specific adaptations in our peer programmes and identify outcomes of our influence in the health system and policy‐making processes. Over time, these insights can inform the confidence of a wide range of stakeholders that the functions are being fulfilled and that effective peer leadership has been demonstrated—confidence which can be monitored quantitatively (e.g. via surveys).

The same approach may be taken to monitor changes in structural stigma over time. Its processes operate and its effects are felt at every level of the socio‐ecological system, from individual lives up to policy and legislation. This means stigma is not “one thing” to measure: it is a constantly moving target, motivated by diverse drivers and facilitators, and manifesting in diverse ways and locations. Instead, a range of indicators must be used, monitoring for impacts on different levels, and drawing insights from a diverse array of stakeholders [40]. Our approach here is consistent with the practices of community monitoring [33], which invite PLHIV and members of key populations to participate in combined internal‐external evaluation of interventions, funding arrangements, policy‐making and healthcare provision.

There are limitations in the applicability of our work to date. The system map and framework have been based on the expertise and experience of participants from 10 PLHIV inclusive peer‐led organizations in Australia, and the examples on the expertise of four PLHIV peer‐led organizations. The participants may have experiences of systemic stigma, healthcare, social and economic opportunities, organizational support and access to policy makers that may not be generalizable to other countries.

The final stage of the project (January 2020–June 2023) is underway, and is consolidating evidence of the system‐level influence of selected peer organizations within the Australia‐wide HIV response.

## 5 CONCLUSIONS

Structural stigma is pernicious and pervasive, even within the organized response to the HIV pandemic. This Australian system mapping study found that effective peer and PLHIV leadership can reduce structural stigma over time, drawing insights from lived experience and practice wisdom to understand and intervene in its processes and effects. However, effective peer leadership is itself affected by structural stigma, and peer leaders and the programmes that support and enable peer leadership must navigate a complex network of causal pathways and strategic pitfalls to demonstrate effectiveness and maintain positive valuation of their work. Participants identified that incorporating PLHIV leadership created a virtuous cycle, because, as positive voices are heard and trusted, the case for their inclusion only gets stronger. A systems perspective can help to guide the most productive points for intervention to tackle structural stigma and promote effective PLHIV leadership.

## COMPETING INTERESTS

GB has received funding from the Australian Government Department of Health, ViiV Healthcare International, Gilead Sciences and the Australian Federation of AIDS Organisations. Other authors have received funding from Australian Government Department of Health and jurisdictional (State) Departments of Health. No pharmaceutical grants were received in the development of this study. AC, BA, CH, JR and DR currently or previously work in PLHIV peer‐led organizations.

## AUTHORS’ CONTRIBUTIONS

GB and DR: Conceptualization, data curation, formal analysis, funding acquisition, investigation, methodology, project administration, visualization, writing—original draft, review and editing. AC, BA and CH: Conceptualization, formal analysis, visualization, validation, writing—review and editing. SC, DG and JR: Validation, writing—review and editing. GB, DR, AC, BA and CH were among the participants in the system mapping and indicator development workshops.

## FUNDING

This study was funded by the Australian Government Department of Health (Blood Borne Viruses and Sexually Transmissible Infections Surveillance Grant).

## Data Availability

The authors confirm that the data supporting the findings of this study are available within the article. Full description of the protocols and methods, and the detailed system maps and their accompanying full text descriptions (the qualitative data analysed in this paper) are available from www.w3project.org.au Further data requests concerning this study can be made by contacting the corresponding author graham.brown@unsw.edu.au.
